# Conventional transarterial chemoembolization versus drug-eluting bead transarterial chemoembolization for the treatment of hepatocellular carcinoma

**DOI:** 10.1186/s12885-015-1480-x

**Published:** 2015-06-10

**Authors:** Roman Kloeckner, Arndt Weinmann, Friederike Prinz, Daniel Pinto dos Santos, Christian Ruckes, Christoph Dueber, Michael Bernhard Pitton

**Affiliations:** 1 Department of Diagnostic and Interventional Radiology, Johannes Gutenberg-University Medical Centre, Langenbeckstr. 1, 55131 Mainz, Germany; 2 Department of Hepatology, Johannes Gutenberg-University Medical Centre, Mainz, Langenbeckstr.1, 55131 Mainz, Germany; 3 Interdisciplinary Center for Clinical Trials (IZKS), Mainz, Germany

**Keywords:** Conventional transarterial chemoembolization (cTACE), Drug-eluting bead transarterial chemoembolization (DEB-TACE), Hepatocellular carcinoma (HCC)

## Abstract

**Background:**

To compare the overall survival of patients with hepatocellular carcinoma (HCC) who were treated with lipiodol-based conventional transarterial chemoembolization (cTACE) with that of patients treated with drug-eluting bead transarterial chemoembolization (DEB-TACE).

**Methods:**

By an electronic search of our radiology information system, we identified 674 patients that received TACE between November 2002 and July 2013. A total of 520 patients received cTACE, and 154 received DEB-TACE. In total, 424 patients were excluded for the following reasons: tumor type other than HCC (n = 91), liver transplantation after TACE (n = 119), lack of histological grading (n = 58), incomplete laboratory values (n = 15), other reasons (e.g., previous systemic chemotherapy) (n = 114), or were lost to follow-up (n = 27). Therefore, 250 patients were finally included for comparative analysis (n = 174 cTACE; n = 76 DEB-TACE).

**Results:**

There were no significant differences between the two groups regarding sex, overall status (Barcelona Clinic Liver Cancer classification), liver function (Child-Pugh), portal invasion, tumor load, or tumor grading (all *p* > 0.05). The mean number of treatment sessions was 4 ± 3.1 in the cTACE group versus 2.9 ± 1.8 in the DEB-TACE group (*p* = 0.01). Median survival was 409 days (95 % CI: 321–488 days) in the cTACE group, compared with 369 days (95 % CI: 310–589 days) in the DEB-TACE group (*p* = 0.76). In the subgroup of Child A patients, the survival was 602 days (484–792 days) for cTACE versus 627 days (364–788 days) for DEB-TACE (*p* = 0.39). In Child B/C patients, the survival was considerably lower: 223 days (165–315 days) for cTACE versus 226 days (114–335 days) for DEB-TACE (*p* = 0.53).

**Conclusion:**

The present study showed no significant difference in overall survival between cTACE and DEB-TACE in patients with HCC. However, the significantly lower number of treatments needed in the DEB-TACE group makes it a more appealing treatment option than cTACE for appropriately selected patients with unresectable HCC.

## Background

Hepatocellular carcinoma (HCC) is one of the most common cancers, with an annual incidence of approximately 750,000 cases per year worldwide [1, 2]. Its incidence continues to increase, mainly because of the increasing incidence of hepatitis B virus (HBV) and hepatitis C virus (HCV) infections [3]. The majority of patients are diagnosed at intermediate or advanced clinical stages, which excludes them from potentially curative treatments such as resection, liver transplantation (LTX), or local ablation. According to the Barcelona Clinic Liver Cancer classification (BCLC), transarterial chemoembolization (TACE) is the standard of care for patients with intermediate stage HCC (BCLC stage B) [1, 4, 5].

Drug-eluting bead transarterial chemoembolization (DEB-TACE) has been widely commercially available since 2006. Since then, DEB-TACE has become the de facto standard in many centers worldwide; numerous investigators believe it to be more beneficial than conventional TACE with lipiodol (cTACE) [6]. Some authors recently reported median survival times of more than 4 years after DEB-TACE in well selected cohorts [7]. Nonetheless, evidence is limited regarding the direct comparison of DEB-TACE and cTACE. Only four studies were conducted in a randomized, prospective fashion [8–11], and none of them showed a significant improvement of a hard endpoint. All other trials were retrospective and mostly based on small patient samples or were lacking the assessment of hard endpoints [12–17]. Two meta-analyses have been published so far. Although they were based on nearly the same studies, their results were inconsistent. The first, published in 2013, found no difference in tumor response between cTACE and DEB-TACE [18]. The second was published in 2014, and found that DEB-TACE provided better tumor response and better overall survival (OS) at 1 and 2 years; however, OS did not differ between the two methods at 6 months or at 3 years [19]. Therefore, the purpose of the present study was to compare the OS of a relatively large cohort of patients with HCC treated with cTACE and DEB-TACE, taking into account histological tumor grading, overall status, liver function, and tumor load.

## Methods

### Study details and data acquisition

The present study is a retrospective, single-center, non-randomized trial in which two parallel treatment groups received either DEB-TACE or cTACE. The trial was conducted based on the principles of the International Conference on Harmonisation of Good Clinical Practice guidelines and according to the Declaration of Helsinki in its revised version. Institutional review board approval was waived by the responsible Ethics Committee of Rhineland Palatinate, Germany given the retrospective study design and analysis of clinical data. Patient records and information were anonymized and de-identified prior to analysis. The study included data from the doctoral thesis of one of the authors (FP).

**Fig. 1:**
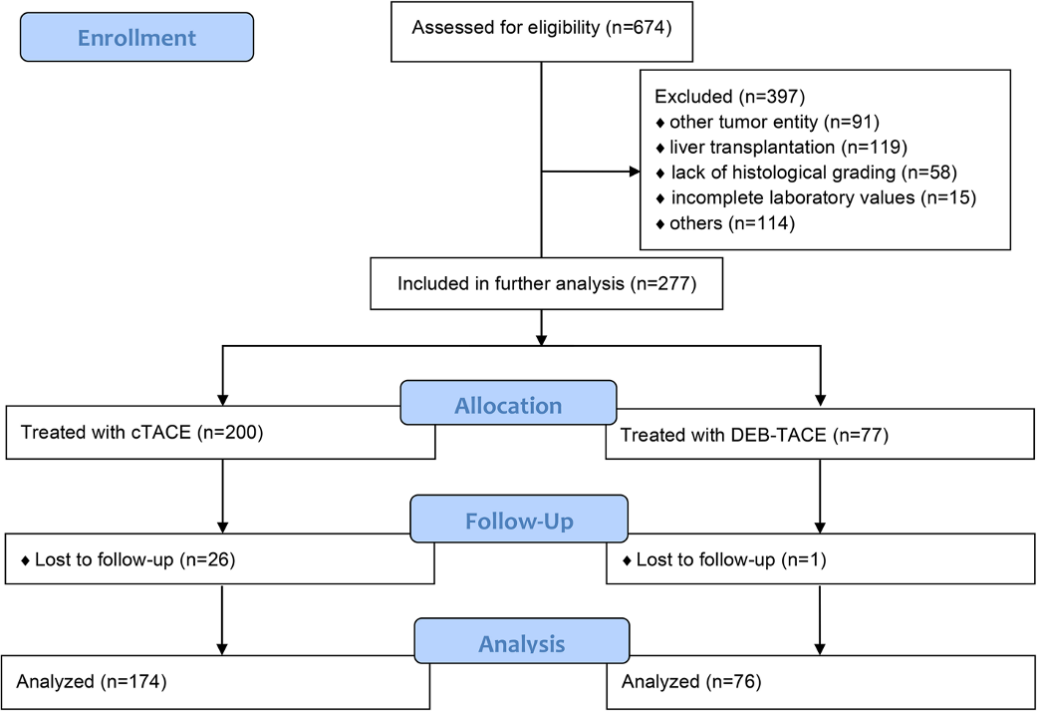
CONSORT flow diagram

Our electronic radiology information system (RIS) was searched to identify all patients treated with TACE at our institution between November 2002 and July 2013, and thus, 674 patients were included. The treatment of the last included patient started in June 2012. All events until the final evaluation date, 22 December 2013, were recorded; therefore, the minimum follow-up period was 1.5 years. A total of 520 patients received cTACE, and 154 received DEB-TACE. In total, 397 patients were excluded because they had a tumor entity other than HCC (n = 91), liver transplantation after TACE (n = 119), lack of histological grading (n = 58), and incomplete laboratory values (n = 15). Additionally, 114 patients were excluded for other reasons as follows: essential data missing in the patient record (n = 45), previous systemic chemotherapy (sorafenib, erlotinib or others; n = 27), previous cisplatin based TACE (n = 13), diffuse tumor (not measurable; n = 10), previous selective internal radiation therapy (SIRT) (n = 8), previous bland embolization (n = 6), abortion of first TACE session (n = 2), previous irinotecan loaded DEB-TACE (n = 2), participation in SPACE study (therefore the patient received either additional sorafenib or placebo treatment; n = 1). Of the remaining 277 patients, 27 were lost to follow-up. Therefore, 250 patients were finally included for comparative analysis (n = 174 cTACE; n = 76 DEB-TACE) (Fig. 1).

### Patient stratification

Overall status, initial liver function, and tumor burden are known to significantly affect the outcome of patients with HCC after treatment [4, 20–22]. Herein, overall status was represented by the BCLC classification, initial liver function by the Child-Pugh score, and tumor burden by the sum of the longest diameter (SLD) of the target lesions and the status according to the Milan criteria (in or out). Additionally, patients were stratified by histological grading, which appears to have considerable impact on survival after liver transplantation [23–25] and TACE [11]. Patients who underwent resection or local ablative therapy after TACE were excluded. To further eliminate any bias, patients who underwent liver transplantation were excluded as this procedure usually leads to a considerably longer OS.

### Treatment

Our interdisciplinary tumor board (Diagnostic and Interventional Radiology, Hepatobiliary/Transplant Surgery, Hepatology) discussed the indication for TACE treatment for each patient. Patients were scheduled consecutively for admission as inpatients. Each pretherapeutic workup included computed tomography (CT) or magnetic resonance imaging (MRI), up-to-date blood laboratory test results, and clinical examination. Treatment was scheduled to take place the next day. TACE was conducted by injecting the embolizing agent (10 mg Mitomycin C [Mito-Medac®; Medac, Hamburg, Germany] emulsified with 10 ml iodized oil [Lipiodol Ultra-Fluide®; Guerbet Laboratories, Aulnay-Sous-Bois, France] or polyvinyl alcohol particles [DcBeads® 500-700 μm, 300-500 μm, and 100-300 μm; Biocompatibles, Farnham, UK; loaded with 150 mg doxorubicin]) into the tumor-supplying vessels as described elsewhere [8, 26]. Embolization was stopped early in case of sluggish flow. Additional bland embolization was not performed. After treatment, all patients received a control CT on the same or the next day to rule out extrahepatic displacement of embolization material and other complications. If no post-interventional complications occurred, patients were discharged 1 or 2 days after treatment, depending on their clinical condition. TACE was repeated every 6 weeks until no more viable tumor was detected by CT or MRI [8, 10] or until any contraindications occurred.

### Image analysis and patient status

The SLD was calculated according to mRECIST criteria [27]. BCLC and Child-Pugh stages were calculated for each patient on the basis of pre-interventional cross-sectional imaging, respective blood test results, and documented clinical investigations [4, 21].

### Statistical analysis

Time-to-event data were analyzed using the Log-rank test and the Kaplan-Meier method, and descriptive statistics of all other parameters were provided. The null hypothesis was that there was no difference in OS for patients with HCC treated with cTACE versus DEB-TACE. The date of first treatment was applied to calculate OS. A proportional hazards model was applied to all variables to identify independent predictors of survival and to calculate hazard ratios with their corresponding 95 % confidence intervals. Baseline characteristics were compared using chi-squared tests and *t*-tests, depending on the scale level. Number of TACE sessions and duration were analyzed using *t*-tests. Statistical Analysis System (SAS®), version 9.2 (SAS Institute Inc., Cary NC, USA) was used for analysis. Kaplan-Meier curves were drawn using SPSS Statistics®, version 22 (IBM, Armonk, USA). An independent statistician performed statistical testing to avoid review bias (CR).

## Results

There were no significant differences between the treatment groups regarding sex, overall status (BCLC), portal invasion, liver function (Child-Pugh), tumor load, or tumor grading (all *p* > 0.05; Table 1). The only significant differences between the two groups were for the distribution of patients younger than 60 years (26.4 % vs. 14.5 % of patients; *p* = 0.04), cryptogenic liver cirrhosis (2.9 % vs. 10.5 % of patients; *p* = 0.01) and prior curative treatment (9.8 % vs. 22.4 % of patients; *p* = 0.01) for cTACE and DEB-TACE, respectively.

**Table 1:** Baseline characteristics of both groups

	cTACE (n = 174)		DEB TACE (n = 76)		
	n	%	n	%	p
Patient demographics					
Sex					0.17
Male	144	82.8	68	89.5	
Female	30	17.2	8	10.5	
Age (years)					0.04
<60	46	26.4	11	14.5	
≥60	128	73.6	65	85.5	
Etiology of liver cirrhosis^a^					
Alcohol					0.15
Yes	86	49.4	30	39.5	
No	88	50.6	46	60.5	
HCV					0.91
Yes	47	27.0	20	26.3	
No	127	73.0	56	73.7	
HBV					0.21
Yes	14	8.0	10	13.2	
No	160	92.0	66	86.8	
NASH					0.29
Yes	10	5.7	2	2.6	
No	164	94.3	74	97.4	
Cryptogen					0.01
Yes	5	2.9	8	10.5	
No	169	97.1	68	89.5	
Prior curative treatment					0.01
Yes	17	9.8	17	22.4	
No	157	90.2	59	77.6	
Liver function/patient status					
BCLC					0.21
A	30	17.2	8	10.5	
B	59	33.9	34	44.7	
C	77	44.3	30	39.5	
D	8	4.6	4	5.3	
ECOG					0.44
0	110	63.2	53	69.7	
1	61	35.1	20	26.3	
2	2	1.1	2	2.6	
3	1	0.6	1	1.3	
Portal invasion					0.25
Yes	36	20.7	11	14.5	
No	138	79.3	65	85.5	
Metastasis					0.66
Yes	5	2.9	3	3.9	
No	169	97.1	73	96.1	
Child					0.48
A	103	59.2	51	67.1	
B	64	36.8	22	28.9	
C	7	4.0	3	3.9	
Bilirubin (mg/dl)					0.40
<2	136	78.2	64	84.2	
2-3	24	13.8	9	11.8	
>3	14	8.0	3	3.9	
Albumin (mg/dl)					0.54
>3.5	76	43.7	29	38.2	
2.8-3.5	67	38.5	35	46.1	
<2.8	31	17.8	12	15.8	
INR					0.35
<1.7	172	98.9	76	100.0	
1.7-2.3	2	1.1	0	0.0	
>2.3	0	0.0	0	0.0	
Ascites					0.55
None	118	67.8	61	80.3	
Mild	38	21.8	11	14.5	
Moderate to severe	18	10.3	4	5.3	
Hepatic encephalopathy					0.26
None	173	99.4	75	98.7	
Grade I-II	1	0.6	1	1.3	
Grade III-IV	0	0.0	0	0.0	
Tumor characteristics					
Tumor grading					0.08
G1	80	46.0	34	44.7	
G2	82	47.1	30	39.5	
G3	12	6.9	12	15.8	
Milan criteria					0.13
In	47	27.0	14	18.4	
Out	127	73.0	62	81.6	
SLD (cm)					0.93
<3	19	10.9	8	10.5	
≥3	155	89.1	68	89.5	

*HBV* hepatitis B virus, *HCV* hepatitis C virus, *NASH* nonalcoholic steatohepatitis, *BCLC* barcelona clinic liver cancer classification, *ECOG* eastern cooperative oncology group, *INR* international normalized ratio, *SLD* sum of the longest diameter

^a^5 patients with HBV/HCV co-infection

The mean number of treatment sessions was 4.0 ± 3.1 in the cTACE group versus 2.9 ± 1.8 in the DEB-TACE group (*p* = 0.01; Table 2). Consequently, the total duration of TACE treatment was significantly longer in the cTACE group than in the DEB-TACE group (217.4 ± 266.1 days vs. 143.9 ± 171.5 days, respectively; *p* = 0.01). A total of 97 patients had subsequent treatment after the cessation of TACE treatment: 69 in the cTACE group (40 %) and 28 in the DEB-TACE group (37 %) (Table 2). After cTACE, the 69 patients received secondary treatment with DEB-TACE (n = 27; 16 %), SIRT (n = 11; 6 %), local ablation (n = 12; 7 %), surgery (n = 5; 3 %), sorafenib (n = 25; 14 %), or other systemic drugs (n = 31; 18 %); 16 of these patients received two or more of these treatments. A total of 105 patients (60 %) received no secondary treatment and either died during TACE treatment or received best supportive care. After cessation of DEB-TACE, 15 patients (20 %) received secondary treatment with cTACE, SIRT (n = 1; 1 %), local ablation (n = 2; 3 %), surgery (n = 1; 1 %), sorafenib (n = 12; 16 %), or other systemic drugs (n = 12; 16 %); 7 of these patients received two or more of these treatments. Forty-eight patients (63 %) received no secondary treatment (Table 2). In summary, subsequent treatments did not differ significantly between the two groups (*p* > 0.05 in all categories).

**Table 2:** TACE treatment characteristics and subsequent treatments

	cTACE (n = 174)		DEB TACE (n = 76)		p
TACE treatment	mean ± SD (range)		mean ± SD (range)		
TACE sessions per patient (n)	4.00 ± 3.09 (1–18)		2.96 ± 1.79 (1–9)		<0.01
Total duration of TACE treatment (days)	217.4 ± 266.1		143.9 ± 171.5		0.01
Subsequent treatment	n^a^	%	n^a^	%	
None	105	60.3	48	63.2	0.54
Crossover to other type of TACE					0.41
Yes	27	15.5	15	19.7	
No	147	84.5	61	80.3	
SIRT					0.09
Yes	11	6.3	1	1.3	
No	163	93.7	75	98.7	
Local ablation					0.18
Yes	12	6.9	2	2.6	
No	162	93.1	74	97.4	
Surgery					0.46
Yes	5	2.9	1	1.3	
No	169	97.1	75	98.7	
Sorafenib					0.77
Yes	25	14.4	12	15.8	
No	149	85.6	64	84.2	
Other systemic therapy					0.70
Yes	31	17.8	12	15.8	
No	143	82.2	64	84.2	
Patients receiving ≥2 treatments					1.00
Yes	16	9.2	7	9.2	
No	158	90.8	69	90.8	

*TACE* transarterial chemoembolization, *SD* standard deviation, *SIRT* selective internal radiotherapy

^a^Some patients received ≥2 subsequent treatments; therefore, the total number of treatments is greater than the total number of patients

**Fig. 2:**
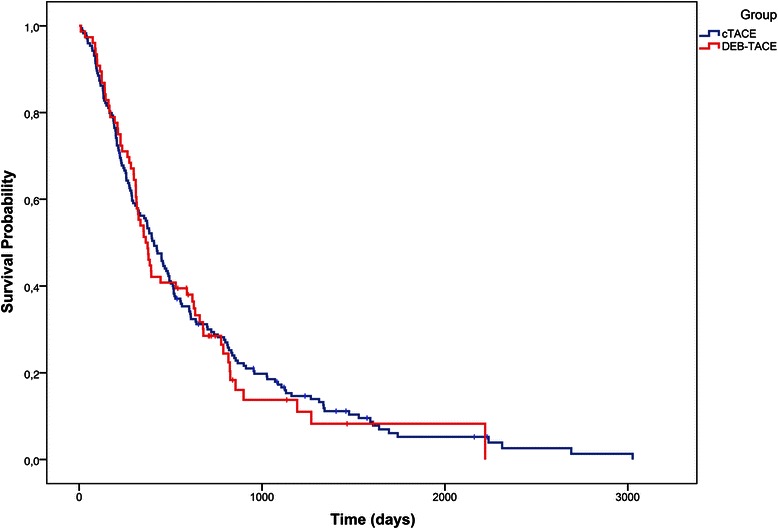
Overall survival: cTACE vs. DEB-TACE (*p* = 0.76). “I” denotes censored values

**Fig. 3:**
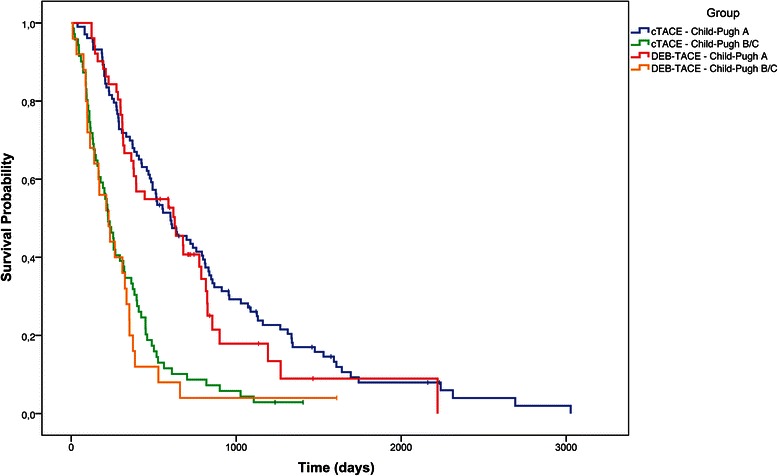
Overall survival: group 1 (cTACE) vs. group 2 (DEB-TACE), differentiated by Child stages A and B/C. *P*-values were 0.4 for Child A and 0.53 for Child B/C patients. “I” denotes censored values

**Fig. 4:**
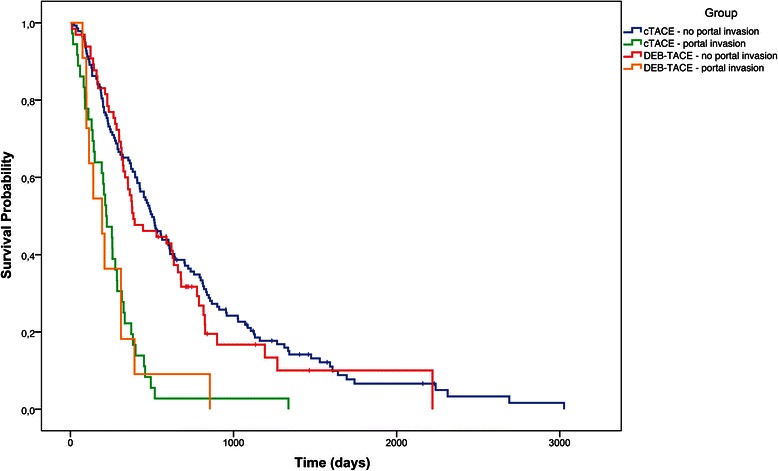
Overall survival: group 1 (cTACE) vs. group 2 (DEB-TACE), differentiated by status of portal invasion. *P*-values were 0.82 for patients with and 0.48 for patients without portal invasion. “I” denotes censored values

Median survival in the cTACE group was 409 days (95 % CI: 321–488 days), compared with 369 days (95 % CI: 310–589 days) in the DEB-TACE group (Fig. 2 and Table 3; *p* = 0.76). The proportional hazards model revealed that Child-Pugh stage and portal invasion were the only independent predictors of survival (Table 4). Therefore, we conducted an additional analysis of the treatment effects according to Child-Pugh stage and portal invasion. In the subgroup of Child A patients, the median OS was 602 days (95 % CI: 484–792 days) for cTACE versus 627 days (95 % CI: 364–788 days) for DEB-TACE (Fig. 3; *p* = 0.40). Because of the small number of Child C patients (n = 7 in the cTACE group and n = 3 in the DEB-TACE group), Child stages B and C were combined for further analysis. The OS was considerably lower in Child B/C patients: 223 days (95 % CI: 165–315 days) for cTACE versus 226 days (95 % CI: 114–335 days) for DEB-TACE (Fig. 3; p = 0.53). As expected, patients with portal invasion exhibited significantly shorter OS than patients without portal invasion in each group (Fig. 4; *p* < 0.01). Nonetheless, when the subgroups of patients with and without portal invasion were analyzed separately, cTACE and DEB-TACE performed equally well (Table 3). The OS in the subgroup with portal invasion was 221 days (95 % CI: 143–285 days) in patients treated with cTACE versus 194 days (97–310 days) in patients treated with DEB-TACE (*p* = 0.82). In patients without portal invasion, OS was 501 days (410–607 days) for cTACE compared with 386 days (325–634 days) for DEB-TACE; p = 0.48.

**Table 3:** OS of all patients, Child A and Child B/C subgroups and patients with/without portal invasion

	cTACE (n = 174)	DEB-TACE (n = 76)	p
OS (days)	median (CI)	median (CI)	
All patients	409 (321–488)	369 (310–589)	0.76
Child A	602 (484–792)	627 (364–788)	0.40
Child B/C	223 (165–315)	226 (114–335)	0.53
Portal invasion			
Yes	221 (143–285)	194 (97–310)	0.82
No	501 (410–607)	386 (325–634)	0.48

*OS* overall survival, *CI* confidence interval, *DEB-TACE* drug-eluting bead transarterial chemoembolization, *cTACE* conventional TACE

**Table 4:** Proportional hazards model to identify independent predictors of survival

Potential predictors of survival	p
Treatment group	0.31
Sex	0.31
Tumor grade	0.09
Child	<0.01
ECOG	0.19
Unilobar vs. bilobar tumor	0.07
Number of nodules	0.09
Portal invasion	<0.01

*ECOG*: eastern cooperative oncology group

## Discussion

Since 2006, when the first drug-eluting beads became commercially available, DEB-TACE has become the de facto standard in many centers, gradually replacing lipiodol-based cTACE as the standard treatment for patients with inoperable HCC. Nonetheless, the scientific basis for that paradigm shift is poor. The PRECISION V trial was by far the biggest study to compare TACE with DEB-TACE, including 212 patients [8]; its primary endpoint was tumor response measured by MRI after 6 months. However, the lack of hard endpoints, such as OS or progression-free survival (PFS), was criticized afterwards [28]. At least some benefit was shown for DEB-TACE over cTACE regarding objective response in the subgroup of more advanced patients (Child-Pugh B, ECOG 1, bilobar disease, and recurrent disease). Reyes et al. published another prospective randomized trial in 2009 [9]. Unfortunately, this phase II trial included only 20 patients, too few to allow a definitive assessment of the secondary endpoints, PFS and OS. Sacco et al. published a series of 67 patients in which they primarily investigated safety, toxicity, and tumor response after 1 month; survival only served as a secondary endpoint [10]. The study by Van Malenstein et al. was also restricted regarding the evaluation of toxicity and safety; tumor response was measured only once 6 weeks after the first treatment [11]. All other available studies were retrospective [12–17]. Three of them relied primarily on surrogate endpoints based on cross-sectional imaging [13, 14, 16], and two of these reported OS as a secondary endpoint [13, 14]. One retrospective trial only reported complications [29]. Only the studies by Dhanasekaran et al., Scartozzi et al., and Wiggermann et al. [12, 15, 17] primarily analyzed hard endpoints such as PFS or OS. Nonetheless, the results of these three studies differed considerably. Dhanasekaran et al. and Wiggermann et al. each reported significant survival benefits of DEB-TACE, while the results of Scartozzi et al. indicated the opposite. Notably, none of these studies considered tumor grading, although it has been reported to have a significant impact on the outcome of patients with HCC after liver transplantation [23–25], and TACE [11].

Llovet et al. and Lo et al. were able to demonstrate a survival benefit of TACE versus symptomatic treatment in 2002 [30, 31]. Nonetheless, in 2011 this statement in general was heavily attacked by the Cochrane Review, which concluded that a clear survival benefit of any type of TACE has not yet been demonstrated [32]. Hence, we decided to employ OS as a primary endpoint and believe that future prospective studies should also be based on hard endpoints such as OS or PFS [5, 33].

Altogether, the present study showed no significant difference in OS between cTACE and DEB-TACE. Additional analyses were conducted to take into account Child-Pugh stages and the status of portal invasion; both methods led to comparable survival times. During the preparation of our manuscript, a prospective analysis was published. This trial was stopped early for futility and found no significant difference between cTACE and DEB TACE in the 2-year survival – further supporting our OS data presented herein [34]. The main difference in our study was the significantly lower number of treatments needed in the DEB-TACE group compared with cTACE, which is likely to enhance patient comfort. We consider that these findings are highly relevant for clinical decision-making. Patients can be exposed to a lower number of treatment sessions, thus lowering their risk of procedure-associated complications. Additionally, the need for less treatment sessions makes DEB-TACE more cost efficient than cTACE despite the considerable price difference between lipiodol and drug-eluting beads.

The main weakness of this study is its retrospective, single-centered and non-randomized design. We tried to eliminate any bias by defining clear drop-out criteria before analysis, which led to the exclusion of 63 % of patients (Fig. 1). The remaining cTACE and DEB-TACE cohorts exhibited no significant difference in any of the factors known to significantly affect outcome. A relatively high percentage of patients were classified as BCLC C. According to the BCLC scheme, stage C patients should receive sorafenib therapy. Nonetheless, this is not reflected in real clinical practice in most major liver disease centers worldwide. Especially if a patient is classified as BCLC C because of having ECOG stage 1, TACE is often preferred over sorafenib. As this was the case in most of our BCLC C patients, we believe that our collective represents the typical TACE patient quite well. Another limitation is the use of two different chemotherapeutic agents. Nonetheless, we believe that this difference is negligible. To this day, no drug or combination of drugs has been proven better than any other for the treatment of HCC in a randomized trial [35]. Only one non-randomized retrospective trial found that cisplatin was superior to doxorubicin, but at the cost of higher side effects [36]. We did not investigate complications as this was not the focus of our study, and this is generally difficult to assess in a retrospective analysis. Furthermore, we used DC Beads® ranging in diameter from 100–300 μm to 500–700 μm. The larger drug-eluting beads were primarily used at the beginning of the treatment period; from 2010 on, we completely switched to smaller beads of 100–300 μm, which we believe preserve the patency of the arterial feeding vessels, thereby allowing more repetitive treatments. From 2012 on, we switched to the even smaller DC Beads M1® (70–150 μm). Nonetheless, it is questionable whether the assumed positive effect of smaller beads might have changed the present results.

## Conclusions

This study showed no significant difference in OS between cTACE and DEB-TACE in a large and comparable cohort of patients with HCC. However, the significantly lower number of treatments needed in the DEB-TACE group might enhance patient comfort, possibly lower the risk of procedure-associated complications among these patients and improve cost-efficiency. Altogether, this makes DEB-TACE a more appealing treatment option than cTACE for appropriately selected patients with unresectable HCC. Given the lack of a significant difference in OS between cTACE and DEB-TACE, researchers seeking to enhance patient OS may consider focusing further efforts on improving the proper selection of patients slated for TACE treatment in general.

## Abbreviations

HBV: Hepatitis B virus
HCV: Hepatitis C virus
BCLC: Barcelona clinic liver cancer classification
TACE: Transarterial chemoembolization
DEB-TACE: Drug-eluting bead transarterial chemoembolization
cTACE: Conventional transarterial chemoembolization
MRI: Magnetic resonance imaging
OS: Overall survival
PFS: Progression-free survival
LTX: Liver transplantation
RIS: Radiology information system
CT: Computed tomography
SLD: Sum of the longest diameter
SIRT: Selective internal radiation therapy
